# Systematization of nursing care as a professional practice model in nursing in Brazil: a critical-epistemological proposition

**DOI:** 10.1590/1980-220X-REEUSP-2025-0551en

**Published:** 2026-06-26

**Authors:** Rudval Souza da Silva, Marcos Antônio Gomes Brandão, Alba Lúcia Bottura Leite de Barros, Marcia Regina Cubas, Jackeline Felix de Souza, Ana Raquel Lima Peralva de Almeida, Laura Emmanuela Lima Costa

**Affiliations:** 1 Universidade do Estado da Bahia, Programa de Pós-graduação em Ciências do Cuidar e Saúde, Senhor do Bonfim, BA, Brazil.; 2 Universidade Federal do Rio de Janeiro, Escola de Enfermagem Anna Nery, Rio de Janeiro, RJ, Brazil.; 3 Universidade Federal de São Paulo, Escola Paulista de Enfermagem, São Paulo, SP, Brazil.; 4 Pontifícia Universidade Católica do Paraná, Programa de Pós-Graduação em Tecnologia em Saúde, Curitiba, PR, Brazil.; 5 Universidade Federal da Bahia, Programa de Pós-graduação em Enfermagem e Saúde, Salvador, BA, Brazil.; 6 Universidade do Estado da Bahia, Jacobina, BA, Brazil.

**Keywords:** Nursing Care, Professional Practice, Nursing Process, Organization and Administration

## Abstract

**Objective:**

To analyze the historiography of the concept of Systematization of Nursing Care (*SAE*) as a Professional Practice Model in Nursing in Brazil, improving the understanding of its conceptual and operational limitations.

**Method:**

A theoretical and reflective essay, grounded in documentary research. Regulatory acts were examined in a proposed analysis of the concept of *SAE*, in addition to reference books and indexed articles, composing *SAE* historiography in Brazil and its interfaces with the Nursing Process. The analysis was conducted through analytical reading and narrative synthesis.

**Results:**

The structural pillars of the *SAE* concept, “method”, “personnel,” and “tools,” closely correspond to the essential components of the Professional Practice Models described in the international literature.

**Conclusion:**

It is concluded that the *SAE*, despite its history marked by conceptual ambiguities, remains a significant construct in the organization of professional nursing practice in Brazil. It is considered a Professional Practice Model; aligned with its pillars, it allows for its reinterpretation not as a synonym for the Nursing Process, but as an expanded model of care management within the macropolitical dimension of planning, networking, and the Brazilian Public Health System (*SUS*) policies.

## INTRODUCTION

Nursing, in a historical and global context, is configured as a scientific discipline and a professional field whose practice is based on the promotion of health, safety for the continuity of care, as well as on the management and leadership of health organizations and systems^(1)^. Thus, it achieves a state of consolidated discipline, with its own body of knowledge supported by science^(2)^. In light of this, to achieve their goals, professionals in the field strive to provide care based on evidence-based practice, considering human responses and basic needs, whether affected or not, that sustain life, through a method known worldwide as the Nursing Process – NP, an adaptation of the scientific method to their disciplinary field^(1,2)^.

Based on the contributions of Wanda de Aguiar Horta, the NP reached the Brazilian nursing scene in the last years of the 1970s. Her perspective on the urgency of the NP can already be observed in the preface of the work “*Processo de Enfermagem* (Nursing Process)”, which gained notoriety in the country by stating that “professional autonomy will only be acquired when the entire class begins to use the scientific methodology in its actions, which will only be achieved through the systematic application of the NP”^(3)^.

Since then, various publications of research results have demonstrated the consolidation of the NP in the Brazilian context. Alongside this progress, there has been an observed misuse of the synonymy between the terms “NP” and “systematization of nursing care – *SAE*”.

In an unsystematic search of the Brazilian Journal of Nursing, the first journal in the field in Brazil, dating from 1932, it was possible to observe that in the 1980s the term “methodology of nursing care – MAE” was used; in the following decade the use of the term “NP” is significant and from the year 2000 onwards, the use of the term “*SAE*” appears in the regulations of the Cofen/Regional Councils System, which is sometimes used as a management system, sometimes as a synonym for NP. In this study, we understand them as distinct terms that require a conceptual comprehension for their applicability in the theoretical-practical field.

This conceptual problem has, up to the time of this study, led to the publication of three Resolutions by the Federal Nursing Council. This allows us to consider that reflection is required, and for that purpose, it is important to know the historiography of these concepts, especially the concept of “*SAE*” coined by Brazilian nurses and disseminated throughout this continental Brazil, which is the object of this theoretical reflection.

At the end of the first decade of the 21st century, a study was conducted that sought to identify the currents of thought on *SAE*, identifying three conceptions of thought. The first one is that there is a conceptual difference between the terms *SAE*, *MAE*, and NP. A second subgroup views the terms NP and *MAE* as equivalent, and a third one understands all three terms to be synonymous^(4)^. It is important to highlight that Cofen Resolution No. 358/2009^(5)^, revoked with the publication of Cofen Resolution No. 736/2024^(6)^, and the authors of this reflective essay understand that these are distinct concepts.

That being said, we believe it is relevant to analyze the historiography of the SAE concept, with a focus on the Professional Practice Model. Furthermore, this study seeks to understand the current historical context and whether this epistemological branch has a place in Brazilian nursing within the scope of care management, with a view to supporting its appropriate use by professionals in the nursing and health fields, to the extent that management and organizational thinking on the *SAE* and its alignment with the NP sustains theoretical and methodological foundations for nursing care management. It is also expected that progress will be made in addressing the need to clarify concepts to facilitate understanding. Given that *SAE* is an expression predominantly used in Brazilian publications, it becomes essential for Brazilian researchers to exhaust conceptual and operational questions on the subject, thus advancing in its understanding.

After this brief contextualization and problematization, it is up to us to ask, and thereby broaden our reflections: when we use the expression *SAE*, what is our understanding? How did this expression emerge? Is this a concept that should be abandoned? Is it no longer able to explain the observed phenomena? Or, by making it obsolete, are we not delegitimizing a cultural subjectivity of an expression so deeply rooted in scientific production and in the practice of Brazilian nursing?

In an attempt to create the conditions for such an analysis and present possible epistemological reflections on these questions, it is considered essential to present a historiography of the *SAE* concept in Brazil, discussing the elements that support it to reinforce distinctions and reiterate the concept of NP as a methodological instrument for thinking about, planning, executing, and evaluating professional care that reflects the practice of the nursing team. However, the analysis is further justified by the possibilities of articulating the NP with a Brazilian Professional Practice Model in Nursing, the *SAE*, as a model for the management of nursing care that goes beyond the scope of direct care, allowing for the evaluation of the efficiency and effectiveness of the actions developed, as well as supporting managerial and political decisions focused on the qualification and excellence of the care provided^(7)^.

It is important to highlight the conceptual differences between administration^(8)^ and management^(9)^ of nursing care, as a national construct, resulting from the epistemological constitution of a national literary corpus on the nursing work process, with a view to integrating these dimensions into the nurse’s practice. Care administration is constituted from a macro-political action of the nurse in the Health Care Network of the Brazilian Public Health System (*SUS*), guiding the mobilization of resources and teams. *SAE* organizes and improves the work process of the nursing team, seeking to guarantee comprehensive care and the effective realization of the right to health. Care management, in turn, corresponds to the micropolitical dimension of nursing practice, centered on the execution and coordination of the nursing process in the daily routine of health services. It is based on interdisciplinary teamwork, ensuring the quality and continuity of nursing care, not acting in isolation, but with professionals from the nursing team, as well as with professionals from other teams^(10)^.

Thus, the objective of this theoretical-reflective study was to analyze the historiography of the concept of *SAE* as a Professional Practice Model in Nursing in Brazil, improving the understanding of its conceptual and operational limitations.

### Historiography of SAE In Brazil

Through documentary research, it was possible to identify the first widely disseminated Brazilian publication that may have expanded a concept of systematizing professional practice in Brazil: Horta’s book “Processo de Enfermagem”^(3)^. It is pointed out that this work has become more widely disseminated because Wanda de Aguiar Horta had previously published scientific articles about the functions of nursing and the challenges of organizing practice through the determination of nursing diagnoses, and the development and execution of care plans^(11)^; proposal of the NP methodology^(12)^; development of scientific nursing theories and terminology and of a care plan^(13)^, among others.

Subsequently, regulatory documents from the professional councils are published, with particular emphasis on those from the Regional Nursing Council of São Paulo^(14)^ and Bahia^(15)^, which subsequently gave rise to Cofen Resolution No. 272/2002^(16)^, revoked, as was Cofen Resolution No. 358/2009^(5)^, and having as its current regulatory framework Cofen Resolution no. 736/2024^(6)^. It is worth noting that this last document makes does not mention the *SAE* concept.

Throughout the historical development of Nursing, it can be observed that from the 1960s onwards, there was an intensified interest in Brazil in organizing and grounding professional practice on theoretical and methodological bases, in line with the international movement of NP. However, it was only in the 1980s that the expression “SAE” began to appear in scientific publications, more effectively in Brazilian literature, often used as a synonym for NP^(17)^. This semantic-conceptual migration led to the trend of conceptual use and overlapping addressed in works that aimed to clarify the subtle lines of conceptual differentiation between the terms^(4,17)^.

Again, the normative character moves towards seeking a synthesis of academic discussions. Thus, the regulatory framework, issued by Cofen with Resolution No. 358/2009, provides in its considerations a definition for SAE as a resource that “organizes professional work in terms of method, personnel and instruments, making the operationalization of the NP possible”^(5)^. This same definition is found in a book^(18)^ published in 2006, titled *Teoria e Método em Assistência de Enfermagem,* and already provoked reflection on this conceptual entanglement, acknowledging that they are distinct concepts. Considering that Resolution No. 358/2009 does not provide the adopted references and, taking into account that the definition of *SAE* contained in the aforementioned resolution is *verbatim* what the author defines as *SAE* ^(18)^, it is possible to infer that this may have been a reference adopted by the group that drafted the aforementioned 2009 resolution.

This Resolution, in a way, divides the academic community into two central understandings. The first is that *SAE* would have, among its unique attributes, elements of nursing management and administration^(17)^; and the second is that it is an inappropriate use of the NP, with the latter being the one that should be used^(19)^.

In 2024, Cofen Resolution No. 736/2024 was published, omitting the mention of *SAE*, dealing exclusively with the NP, which is defined as “a method that guides the critical thinking and clinical judgment of the nurse, directing the nursing team towards the care of the individual, family, community and special groups”^(6)^. Based on the aforementioned Resolution, the academic community seems to clearly recognize the distinctive conceptualization between *SAE* and NP in theoretical-conceptual and operational terms, and gives prominence to the NP, which from a pragmatic conceptual perspective was considered the “mature concept”^(17,20)^.

Based on current knowledge, the incorrect use of the terms *SAE* and NP as synonyms negatively impacts the formation of the nurse’s professional identity^(7)^. Upon reflection, it is understood that the suppression of the term *SAE* from Cofen Resolution 736/2024 is a clear, objective, and pragmatic way of highlighting the differences between the two concepts, and that using both as synonyms is a mistake that limits the operational application of the NP. However, based on what historians of the philosophy of science, such as Thomas Kuhn and Imre Lakatos, propose, a paradigm or a program of scientific research and its elements do not usually disappear when questioned^(21)^. Therefore, the core concept of *SAE* should not disappear until it is completely overturned by the lack of operational elements that allow for verification, use, and testing; that is, if it ever disappears.

Starting from the pragmatic conception that SAE is a partially mature concept^(17)^, it has conceptual validity; however, its properties are not entirely refutable. Consequently, it is relevant for the science of the discipline to advance in investigating its structural and operational aspects.

### Advancement of Essential SAE Elements in Brazil

The term “SAE” possibly originates from a proposal by the Nursing Care Division of the University Hospital (HU) of the University of São Paulo (USP). In developing its conceptual framework and objectives in 1981, it defined the goal as “establishing an appropriate system of cooperation and collaboration with physicians and other professionals, aiming at integrated care.” It was given the name “Nursing Care System”. During this period, the terminology underwent modifications, initially being replaced from “Systematics” to “Nursing Care System”^(22)^.

Until then, this system aimed to integrate teaching and care activities as a learning strategy for undergraduate and graduate students, as well as the nursing staff at the University Hospital of USP. In this system, the core guiding principle of professional nursing care is the NP, which sets the standard for the nursing team professionals’ know-how^(22)^. In this context, the Nursing Care System Study Group (GESAE) was created in 1985. The use of the word “system” refers to a set of interrelated elements that interact with each other for a common purpose, forming an organized whole, for data sharing among professionals on the multidisciplinary team^(23,24)^.

Presumably drawing on the experience of the USP’s HU, in the following decade, the Regional Nursing Council of São Paulo published Decision Coren-SP No. 008/1999, which “standardizes the implementation of the *SAE* in health institutions within the State of São Paulo”^(14)^. In this document, the term “system” (a masculine noun in Portuguese) is changed to “systematization” (a feminine noun); however, no explanation is given for this terminological change. This decision was published the following year, with the same text, by the Regional Nursing Council of Bahia – Coren-BA Decision No. 007/2000^(15)^.

In the texts of these Decisions, some points in the recital section allow us to infer that both adopt the expression used by the nurses at HU-USP when using *SAE* in the masculine form in Portuguese (“o SAE”). It also introduces the term “*SAE*,” standardizing it as an “exclusive activity of the Nurse” that “uses scientific work methods and strategies to identify health/illness situations, supporting the prescription and implementation of nursing care actions that can contribute to health promotion, prevention, recovery, and rehabilitation of the individual, family, and community”. Next, it mentions “the institutionalization of the *SAE*,” again in the masculine form, as the practice of a work process suited to the needs of the community and as a care model to be applied in all areas of health care by the Nurse^(14,15)^. Although the feminine form of the word *SAE* is used in other passages, it is inferred that it may originate from the HU System of USP and its organizational and operational meaning. It is worth highlighting that since the first publication in book format by the University Hospital of USP in 1992, the distinction between the NP and the operating system called *SAE* has been clear.

Furthermore, these decisions present a confusing use of the term “NP” as a synonym for “*SAE*,” as stated in their first article: “The Nurse is responsible for: I – exclusively the implementation, planning, organization, execution and evaluation of the NP, which comprises the following stages: Nursing Consultation; History Taking and Physical Examination; Nursing Diagnosis; Nursing Prescription and Nursing Progress Notes^(14,15)^.

Two inconsistencies can be observed, one regarding the determination of the NP as an activity exclusive to nurses, given that there are stages of the process that are, in fact, exclusive, but not entirely. And the other inconsistency lies in the description of the stages, which are internationally recognized as the stages of the NP.

Further on, the text of the decisions establishes deadlines for the implementation of “SAE” and states in its fifth article that: “The implementation of the *SAE* must be formally recorded in the patient/client’s medical record and must include:” Nursing History; Physical Examination; Nursing Care Prescription; Nursing Care Progress Notes; Nursing Report”^(14,15)^.

Only two years later did the Federal Nursing Council publish Cofen Resolution No. 272/2002^(16)^, which is faithful to the Decisions of the Regional Councils of São Paulo and Bahia, except for the dates for monitoring the implementation process, which do not appear in the Resolution. It was added that it is up to each regional office, within their respective jurisdictions, to promote meetings, seminars, and events to provide technical and scientific support to nursing professionals in the implementation of the *SAE* (in the text of the resolution, the feminine form is always used). This movement will lead to an undesirable conceptual overlap between SAE and NP, which will result in the publication of Cofen Resolution No. 358/2009^(5)^.

Although Cofen Resolution No. 358/2009^(5)^ seeks to present the conceptual distinction, it does not descriptively evoke a particular instrumentality for SAE, which would be the methodological instrument. Due to this condition being understood as an aspect of partial maturity^(17)^, the concept of *SAE* remains imprecise and marginalized from the scientific advancement of the discipline.

In 2020, a group of researchers from several Higher Education and Health Institutions in Brazil created the Nursing Process Research Network (RePPE), with the purpose of generating, synthesizing, and sharing knowledge about the nursing process and classifications. In the pursuit of a conceptual clarification of SAE, and in partnership with Cofen, a new working group was formed to reflect on Cofen Resolution No. 358/2009^(5)^, culminating in the publication of Resolution 736/2024^(6)^. Essentially, it defines the NP as a method that guides the nurse’s critical thinking and clinical judgment so that this professional can direct the nursing team in the care of the individual, family, community, and special groups, emphasizing the need to use theories that underpin the method, and detail the steps that make up the NP, namely: Nursing assessment; Diagnosis; Planning; Implementation and Evaluation^(6)^. This latest resolution presents well-defined structural and operational elements for the concept of PE, consistent with a mature concept and verifiable through both qualitative and quantitative evidence^(20)^.

However, the literature has some essential elements that distinguish SAE, since some researchers have always taken a position regarding the distinctive attributes of the concepts. Publications^(17,25)^ attribute properties closely related to the management of nursing services, to organize the work based on its three pillars (method, personnel, instruments), which can be used to help understand the organizational level of nursing services.

Two perspectives can facilitate efforts to mature the theoretical-conceptual and operational knowledge of *SAE* outside the limits clearly established in the NP: that of *Professional Practice Models* (PPM)^(26)^ and that of a model or management tool operating within the framework of clinical reasoning^(27)^.

A PPM is a conceptual framework that provides nursing with a foundation for safe, patient-centered, and high-quality care, indicating, in addition to values, the structures and processes that support nurses in controlling their practice and the care environment^(26,28)^. In its theoretical foundations, PPM typically encompasses: shared governance as a central concept, core values as a basis, application of nursing theories that explain the nature of care and professional performance, and organizational theories, or models^(28)^.

In the conception of a clinical reasoning, somewhat broadened^(27)^, the SAE concept retains its core administration principles. However, this administration is better defined in its operational and structural components, as outlined in the following section.

### Sae between The Professional Practice Models and Clinical Reasoning an Operational and Structural Analysis

Concept Analysis Study on *SAE* ^(17)^ concludes that this is a partially mature concept, guided by multiple definitions and operationalized based on other concepts. From this perspective, it is worth highlighting that the same study^(17)^ identified the first records of the linguistic use of the terms “systematization” and “to systematize” in the 1960s, demonstrating a characteristic of organizing professional practice in favor of adequate conditions for the nurse’s clinical work, in addition to what has already been presented in this theoretical reflection regarding a hypothesis of the origin of the term “SAE” stemming from a proposal by the Nursing Care Division of the University Hospital of USP, with modifications in its trajectory by the time of the Regional Nursing Councils and then the Federal Council publications.

An editorial, written by a researcher with a post-doctoral degree in Nursing Administration, addressing the administration of nursing care, currently called Nursing Administration, points out that it is mainly focused on meeting the needs of selecting and adapting methods of work organization, which the author highlights in parentheses as *SAE* ^(29)^.

Following this common thread, a recent study^(25)^ considers that the *SAE* is not configured as an epistemological object with a convergent connection to direct patient care, but rather as a concept that brings together properties that ensure it a field of knowledge related to the management and/or administration of nursing services, as a model or even a tool for care management^(7)^. From this perspective, *SAE* encompasses dimensions that transcend direct patient care and enables analysis and reflection on the efficiency and effectiveness of the actions developed by the nursing team, with the aim of contributing to decisionmaking in the management and policies of services, aiming for excellence in care.

Recognizing that SAE is a genuinely Brazilian construct, even though it risks being outlawed, it is important to highlight that, in light of the philosophy of science – especially the notion of “scientific research programs” proposed by Imre Lakatos^(21)^, theories and models are not abandoned in the face of initial difficulties or criticism. Conversely, groups that apply such structures as explanations tend to invest in their development and refinement, adjusting them in response to the issues raised. Abandoning the concept of a *SAE* at this point would correspond to giving in to the logic of premature discarding, to the detriment of the maturation of a theoretical-empirical model with the potential to guide and strengthen the management of professional and scientific nursing practice.

As already mentioned, the existence of constructs potentially correlated to *SAE* in other international contexts, such as PPM^(17)^, reiterates the importance of considering the possibilities of a unique model for Brazil. To a certain extent, a PPM has attributes that converge with the fundamentals of what *SAE* would be, presenting itself as an expanded and structuring lens, capable of re-signifying the *SAE*, especially in the proposition of a systemic approach, integrating professional values, care processes, interprofessional relationships, recognition mechanisms and governance models.

The structure of PPMs consists of essential components that define their organization and operation, such as: professional values^(26)^, a theoretical and conceptual basis that supports the model^(28)^, collaborative professional relationships, organization of patient care^(26,28)^, participatory management approach^(26)^, leadership at all levels of practice^(28)^ and mechanisms for recognizing and valuing nurses^(26,28)^.

Furthermore, the PPMs emerge as epistemological devices that grant Nursing an autonomous, deliberative, and reflective space, supported by fundamental values such as dignity, responsibility, and ethical-aesthetic commitment to excellent care^(26,28)^. By establishing a conceptual architecture that articulates knowledge, practice, and professional being, these models not only guide clinical practice but also redefine decision-making processes in the management and policies of nursing services, contributing to the improvement of care environments and the broadening of the discipline’s horizons^(7)^.

*SAE*, as outlined in the literature, is based on the organization of the nursing team’s work^(30)^, structurally articulated around three fundamental pillars – method, personnel and instruments^(25,31)^. These pillars aim to establish a work dynamic guided by its own system, centered on care management^(10)^, with the purpose of structuring and qualifying the *know-how* of the nursing team in daily care.

In this context, it becomes imperative that nursing leaders recognize that the sharing and consolidation of beliefs, values, knowledge, and practices are not merely organizational strategies, but ontological and epistemological foundations for the (re) construction of professional identity^(7)^. By analogy to the PPMs, the *SAE* therefore points to the need for critical thinking, committed to producing meanings that transcend mere technical operationalization and move towards consolidating Nursing as an applied science and a complex social practice.

Viewed from the perspective of a PPM, the *SAE* presents multiple attributes: it acts as a care model, a work method for organizing processes, an organizational device, a management tool, and a way to achieve effective, qualified, and responsive care to health needs^(30)^.

Thus, its epistemological and operational purpose lies in offering guidelines for the implementation of care actions, guided by the NP in the different care management scenarios. It is, therefore, a structuring model or tool available for the professional *know-how*, useful for articulating theory and practice, knowledge and action, individual and collective, being an identity landmark of Brazilian Nursing.

In its structure, we agree with the authors^(25,31)^ by recognizing that the “method” pillar can be understood in light of the professional values of Nursing, anchored in its own epistemology – especially in Nursing Theories and the NP. This, in turn, constitutes the articulating axis of the nurse’s critical thinking and clinical judgment, guiding the nursing team in providing care to individuals, families, communities, and groups in specific situations of vulnerability. Within the institutional context, this pillar is also related to how care is methodologically organized within the service, considering the mission, philosophy, and objectives of the healthcare institution, as well as the definition of care models that guide nursing practice. Thus, the method is materialized in the adoption of theoretical frameworks, in the operationalization of the steps of the NP, and in the way care is planned, executed, and evaluated in the daily routine of services.

The “personnel” pillar encompasses aspects related to workforce sizing, people management, and the training and development of human resources, which are fundamental to supporting a qualified professional practice aligned with healthcare demands^(25,31)^. This dimension includes elements such as the availability and adequate sizing of the nursing team, institutional strategies for training and continuing education, raising awareness among the team about the importance of *SAE*, as well as the development of clinical and managerial skills necessary for the implementation of the Nursing Process (NP). This pillar also includes aspects related to institutional interest and management’s commitment to supporting the implementation of the SAE, ensuring organizational and structural conditions for its effectiveness.

Finally, the pillar “instruments” is structured around a set of normative and operational devices that support the practice of care, such as: nursing manuals, internal service regulations, standard operating procedures (SOPs), care protocols, clinical bundles, evaluation scales, and nursing-specific forms. These instruments not only operationalize care, but also contribute to its standardization, safety, and quality, in accordance with the principles of SAE and respecting the precepts of the patient safety policy. This dimension also includes the printed materials and record-keeping systems used by the nursing team to document the stages of the NP – such as forms for data collection, diagnostic records, planning, prescriptions, and nursing progress notes – as well as clinical assessment tools that consider the characteristics of the clientele served and the units care profile.

Based on the foregoing, it is possible to state that the structural pillars of the *SAE* closely correspond to the essential components of the Professional Practice Models described in the international literature. The “method” pillar incorporates the NP, a central element of clinical practice, and the theoretical frameworks of the discipline capable of providing descriptions, explanations, predictions, and prescriptions about professional phenomena and events. The “personnel” pillar, encompassing people management, team sizing and qualification, converges with the components related to leadership, professional development, and the work environment. The pillar “instruments”, by bringing together protocols, regulations, forms, and other normative devices, interacts with the elements of the PPM that structure governance, standardization of care, and support for clinical decision-making. Thus, *SAE* is understood not only as a technical-operational arrangement, but as an integrated system of epistemological, organizational, and practical foundations, capable of enhancing both clinical reasoning and care management in multiple dimensions.

These correspondences demonstrate that *SAE*, as a genuinely Brazilian construct, can and should be understood as a situated and robust expression of a Professional Practice Model, reaffirming its relevance as an organizational, epistemological, and ethical foundation for nursing care. In this way, the conceptual and model scope of *SAE* is broadened beyond that of an organizational tool, offering potential as a representative structure for the integration of knowledge and practice.

The nurse, as a care manager, must consider the organization of the entire service and direct care planning based on the NP and clinical reasoning – understood as a dynamic process that articulates cognition, metacognition, and decision-making^(27)^. This systemic approach, in theory, promotes excellence in care, capable of extending beyond diagnosis to encompass therapeutic decisions, monitoring, and continuous evaluation.

From a systemic perspective, SAE is strengthened as a care management tool in a broader and more strategic sense, linked to macro-political, planning, results evaluation, and institutional policies decision-making^(8,10)^, structuring the practice based on professional autonomy, technical competence, and commitment to the subjects of care. This understanding requires ongoing staff training, alignment with legal guidelines, and recognition of SAE as an expression of a practice model grounded in professional values and within the scope of nursing science. The articulation between *SAE*, clinical reasoning, and the fundamentals of PPM therefore offers a promising path for the maturation of the *SAE* concept in Brazil, contributing to the theoretical-practical coherence of care, the qualified management of services, and the strengthening of the identity of Nursing as a discipline and professional field.

Although the reflections in this study may improve knowledge, there is a fundamental limitation. The study has limitations in providing specific instrumental aspects for distinct applications of the *SAE*, which is essential to ensure that no new conceptual overlaps are produced or that advances obtained from clarifying the conceptual maturity of the NP are undermined for clinical practice. However, this limitation depends on continuous improvements in future research that can utilize brought and transferable evidence from PPM to the reality of implementing SAE models in Brazil.

## CONCLUSION

In conclusion, despite its history marked by conceptual ambiguities and semantic disputes, *SAE* remains an important construct for organizing the professional practice of Nursing in Brazil. Its recognition as analogous to a Professional Practice Model, aligned with the pillars of “method,” “personnel,” and “tools,” allows it to be reinterpreted not as a synonym for NP, but as a care management model. The correlation between these pillars and PPM essential components reinforces the potential of the SAE in structuring the work of the nursing team in a critical, reflective and integrated way, promoting not only the quality of care, but also the strengthening of the Nursing identity.

Its continued presence in the disciplinary field depends not only on its normative tradition or its cultural roots, but also on its capacity to be reconfigured in light of solid epistemological foundations, evidence-based practice, and the contemporary demands of health services. Reaffirming SAE as a model implies repositioning it as capable of supporting the macro-political dimension of planning, to guide a participatory management approach and marked by the nurse’s leadership to contribute to excellence in nursing care in Brazil through networking and *SUS* policies.

We conclude reaffirming this analysis’s ethical commitment to the intellectual authorship of the sources. The interpretation of scientific and normative productions preserved the integrity of the original ideas, ensuring that the syntheses produced did not distort the cited authors thoughts.

## Data Availability

The entire dataset supporting the results of this study was published in the article itself.
