# Supplementary material for: Systematization of nursing care as a professional practice model in nursing in Brazil: a critical-epistemological proposition
Source: Rev Esc Enferm USP. 2026 Jun 26;60:e20250551. doi: 10.1590/1980-220X-REEUSP-2025-0551en (PMC13322740; doi:10.1590/1980-220X-REEUSP-2025-0551en)
Supplement: Translation

# Translation: Sistematização da assistência de enfermagem como modelo de prática profissional da enfermagem no Brasil: proposição crítico-epistemológica

**DOI:** 10.1590/1980-220X-REEUSP-2025-0551pt

**Authors:** Rudval Souza da Silva, Marcos Antônio Gomes Brandão, Alba Lúcia Bottura Leite de Barros, Marcia Regina Cubas, Jackeline Felix de Souza, Ana Raquel Lima Peralva de Almeida, Laura Emmanuela Lima Costa

**Affiliations:** 1 Universidade do Estado da Bahia, Programa de Pós-graduação em Ciências do Cuidar e Saúde, Senhor do Bonfim, BA, Brasil.; 2 Universidade Federal do Rio de Janeiro, Escola de Enfermagem Anna Nery, Rio de Janeiro, RJ, Brasil.; 3 Universidade Federal de São Paulo, Escola Paulista de Enfermagem, São Paulo, SP, Brasil.; 4 Pontifícia Universidade Católica do Paraná, Programa de Pós-Graduação em Tecnologia em Saúde, Curitiba, PR, Brasil.; 5 Universidade Federal da Bahia, Programa de Pós-graduação em Enfermagem e Saúde, Salvador, BA, Brasil.; 6 Universidade do Estado da Bahia, Jacobina, BA, Brasil.

**Keywords:** Cuidados de Enfermagem, Prática Profissional, Processo de Enfermagem, Organização e Administração

## Abstract

**Objetivo:**

Analisar a historiografia do conceito Sistematização da Assistência de Enfermagem (SAE) como Modelo de Prática Profissional da Enfermagem no Brasil, avançando na compreensão de seus limites conceituais e operacionais.

**Método:**

Ensaio teórico-reflexivo, ancorado em pesquisa documental. Foram examinados atos normativos numa proposta de análise do conceito da SAE, além de livros de referência e artigos indexados, compondo a historiografia da SAE no Brasil e suas interfaces com o Processo de Enfermagem. A análise ocorreu por leitura analítica e síntese narrativa.

**Resultados:**

Os pilares estruturantes do conceito SAE, “método”, “pessoal” e “instrumentos”, guardam estreita correspondência com os componentes essenciais dos Modelos de Prática Profissional descritos na literatura internacional.

**Conclusão:**

Concluise que a SAE, apesar de sua trajetória marcada por ambiguidades conceituais, permanece como um constructo significativo na organização da prática profissional da Enfermagem brasileira. Situa-se como um Modelo de Prática Profissional; alinhado aos seus pilares, permite reinterpretála não como sinônimo de Processo de Enfermagem, mas como um modelo ampliado de gestão do cuidado numa dimensão da macropolítica de planejamento, articulação em rede e políticas do SUS.

## INTRODUÇÃO

A Enfermagem, num cenário histórico e global, configurase como uma disciplina científica e um campo profissional que fundamenta sua prática na promoção da saúde, segurança para a continuidade do cuidado, assim como na gerência e liderança das organizações e sistemas de saúde^(1)^. Dessa maneira, alcança um estado de disciplina consolidada, com um corpo de conhecimentos próprios apoiado na ciência^(2)^. Frente a isso, para atingir seus objetivos, os profissionais da área empenhamse em cuidar, a partir de uma prática baseada em evidências, considerando as respostas humanas e as necessidades básicas afetadas ou não, que sustentam a vida, por meio de um método próprio mundialmente conhecido como Processo de Enfermagem – PE, uma adaptação do método científico ao seu campo disciplinar^(1,2)^.

A partir das contribuições de Wanda de Aguiar Horta, o PE alcança o cenário da Enfermagem brasileira nos últimos anos da década de 1970. Sua perspectiva de premência do PE pode ser observada já no prefácio da obra “Processo de Enfermagem”, que ganhou notoriedade no país ao afirmar que “a autonomia profissional só será adquirida no momento em que toda a classe passar a utilizar a metodologia científica em suas ações, o que só será alcançado pela aplicação sistemática do PE”^(3)^.

Desde então, diferentes publicações de resultados de pesquisas têm demonstrado a consolidação do PE no cenário brasileiro. Concomitante a esse avanço, tem sido observado o uso equivocado da sinonímia entre os termos “PE” e “sistematização da assistência de enfermagem – SAE”.

Numa busca assistemática na Revista Brasileira de Enfermagem, o primeiro periódico da área no Brasil, datando de 1932, foi possível observar que na década de 1980 era utilizado o termo “metodologia da assistência de enfermagem – MAE”; na década seguinte é marcante o uso do termo “PE” e a partir do ano 2000 surge, nas normativas do Sistema Cofen/Conselhos Regionais, o uso do termo “SAE”, que ora é usado como um sistema de gestão, ora como sinônimo de PE. Nesse estudo, entendemos como termos distintos e que requerem uma compreensão conceitual para sua aplicabilidade no campo teórico-prático.

Essa problemática conceitual gerou, até o momento deste estudo, a publicação de três Resoluções por parte do Conselho Federal de Enfermagem. Isso nos permite considerar que é necessário refletir sobre e, para tal, importa conhecer a historiografia desses conceitos, em especial sobre o conceito “SAE” cunhado por enfermeiras brasileiras e disseminado por toda parte, nesse Brasil continental e objeto dessa reflexão teórica.

No final da primeira década do século XXI, foi realizado um estudo que buscou identificar as correntes de pensamento sobre a SAE, identificando três concepções de pensamento. Uma primeira que entende haver uma diferença conceitual entre os termos SAE, MAE e PE. Um segundo subgrupo que vê como equivalentes os termos PE e MAE e um terceiro que compreende os três termos como sinônimos^(4)^. Importante destacar que a Resolução Cofen nº 358/2009^(5)^, revogada com a publicação da Resolução Cofen nº 736/2024^(6)^,e os autores deste ensaio reflexivo têm o entendimento de que são conceitos distintos.

Isso posto, entendemos ser relevante analisar a historiografia do conceito SAE, com um olhar direcionado pelo Modelo de Prática Profissional. Ademais, busca-se compreender o contexto histórico atual e se essa ramificação epistemológica tem espaço na enfermagem brasileira no âmbito da gestão do cuidado, com vistas a apoiar seu adequado uso pelos profissionais do campo da enfermagem e da saúde até onde o pensamento de gestão e organização sobre a SAE e seu alinhamento com o PE sustenta fundamentos teórico-metodológicos para a gestão do cuidado em enfermagem. Também, espera-se avançar frente à necessidade de clarificação dos conceitos em prol de um possível entendimento. Haja vista ser a SAE uma expressão eminentemente aplicada nas publicações do Brasil, torna-se essencial que pesquisadores brasileiros esgotem questionamentos conceituais e operacionais sobre a temática avançando na sua compreensão.

Após essa breve contextualização e problematização, cabenos questionar, e com isso ampliar as reflexões: quando usamos a expressão SAE, qual é o nosso entendimento? Como se originou essa expressão? É um conceito que deve ser abandonado? Ele não mais consegue explicar os fenômenos observados? Ou será que, ao colocá-lo no desuso, não estamos deslegitimando uma subjetividade cultural de uma expressão tão arraigada nas produções científicas e na prática da enfermagem brasileira?

Na tentativa de produzir as condições para tal análise e apresentar possíveis reflexões epistemológicas a estas perguntas, entende-se ser essencial apresentar uma historiografia acerca do conceito SAE no Brasil, discorrendo sobre os elementos que o sustentam com vista a reforçar distinções e reiterar o conceito de PE como instrumento metodológico para pensar, planejar, executar e avaliar o cuidado profissional que reflete a prática da equipe de enfermagem. Entretanto, a análise justifica-se ainda mais nas possibilidades de articulação do PE com um Modelo de Prática Profissional da enfermagem brasileira que é a SAE, enquanto modelo para a gestão do cuidado de enfermagem que ultrapassa o âmbito do cuidado direto, permitindo avaliar a eficiência e a eficácia das ações desenvolvidas, além de subsidiar decisões gerenciais e políticas com foco na qualificação e excelência do cuidado prestado^(7)^.

Cabe destacar as diferenças conceituais entre gestão^(8)^ e gerência^(9)^ do cuidado de enfermagem, enquanto construto nacional, resultantes da constituição epistemológica de um corpus literário nacional sobre o processo de trabalho de enfermagem, com vistas à integração dessas dimensões na atuação do enfermeiro. A gestão do cuidado constitui-se a partir de uma atuação macropolítica do enfermeiro na Rede de Atenção à Saúde do Sistema Único de Saúde (SUS), orientando a mobilização de recursos e equipes. A SAE organiza e qualifica o processo de trabalho da equipe de enfermagem, em busca da garantia da integralidade do cuidado e a efetivação do direito à saúde. Já a gerência do cuidado corresponde à dimensão micro-política da prática do enfermeiro centrada na execução e coordenação do PE no cotidiano dos serviços de saúde. Fundamentase no trabalho em equipe interdisciplinar, assegurando a qualidade e a continuidade do cuidado de enfermagem, não atuando de modo isolado, mas com profissionais da equipe de enfermagem, assim como com profissionais de outras equipes^(10)^.

Assim, o presente estudo teórico-reflexivo tem por objetivo analisar a historiografia do conceito SAE como Modelo de Prática Profissional da Enfermagem no Brasil, avançando na compreensão de seus limites conceituais e operacionais.

### Historiografia da SAE no Brasil

Por pesquisa documental, foi possível identificar a primeira publicação brasileira de maior difusão que, no Brasil, pode ter expandido uma concepção de sistematização da prática profissional, o livro “Processo de Enfermagem” de Horta^(3)^. Aponta-se como maior difusão, pois Wanda de Aguiar Horta já havia publicado anteriormente artigos científicos acerca das funções da Enfermagem e dos desafios de organização da prática por meio de determinação do diagnóstico de enfermagem, elaboração e execução do plano de cuidados^(11)^; proposição da metodologia do PE^(12)^; desenvolvimento de teorias e terminologias científicas de enfermagem e de um plano assistencial^(13)^, dentre outras.

Posteriormente, documentos normativos dos conselhos da profissão são publicados, cabendo destaque para os do Conselho Regional de Enfermagem de São Paulo^(14)^ e da Bahia^(15)^ que deram origem, posteriormente, à Resolução Cofen nº 272/2002^(16)^, revogada assim como a Resolução Cofen nº 358/2009^(5)^, e tendo como marco regulatório atual a Resolução Cofen nº 736/2024^(6)^. Vale destacar que nesse último documento, não há menção ao conceito SAE.

Durante o percurso histórico da Enfermagem, observase que a partir da década de 1960 intensificou-se, no Brasil, o interesse em organizar e fundamentar a prática profissional em bases teóricas e metodológicas, em consonância com o movimento internacional do PE. Contudo, foi apenas na década de 1980 que a expressão “SAE” passou a aparecer nas publicações científicas, de forma mais efetiva na literatura brasileira, muitas vezes utilizada como sinônimo de PE^(17)^. Esta migração semântico-conceitual levou à tendência de utilização e sobreposição conceitual abordada em produções que objetivaram esclarecer as linhas tênues de diferenciação conceitual dos termos^(4,17)^.

Novamente, o caráter normativo caminha para buscar a síntese das discussões acadêmicas. Desse modo, o marco reguatório, expedido pelo Cofen com a Resolução nº 358/2009, traz em seus considerandos uma definição para a SAE como um recurso que “organiza o trabalho profissional quanto ao método, pessoal e instrumentos, tornando possível a operacionalização do PE”^(5)^. Essa mesma definição é encontrada num livro^(18)^ publicado em 2006 cujo título é Teoria e Método em Assistência de Enfermagem e já provocava essa reflexão relacionada a tal imbróglio conceitual, assumindo que são conceitos distintos. Considerando que a Resolução nº 358/2009 não traz as referências adotadas e, levando em conta que a definição de SAE contida na citada resolução é *ipsis litteris* ao que define como SAE a autora^(18)^, é possível inferir que pode ter sido uma referência adotada pelo grupo que construiu a mencionada resolução de 2009.

Esta Resolução de certo modo divide a comunidade acadêmica em dois entendimentos centrais. O primeiro de que a SAE teria em seus atributos exclusivos, elementos de gestão e administração de enfermagem^(17)^; e a segunda de que ela é um uso inapropriado do PE, sendo este último o que deve ser empregado^(19)^.

Em 2024, a Resolução Cofen nº 736/2024 é publicada omitindo a menção a SAE, tratando exclusivamente do PE que é definido como “um método que orienta o pensamento crítico e o julgamento clínico do enfermeiro direcionando a equipe de enfermagem para o cuidado à pessoa, família, coletividade e grupos especiais”^(6)^. A partir da mencionada Resolução, a comunidade acadêmica parece reconhecer claramente a conceitualização distintiva entre SAE e PE em termos teórico-conceituais e operacionais, e confere protagonismo ao PE, o que na perspectiva pragmática conceitual foi considerado o “conceito maduro”^(17,20)^.

No estado do conhecimento atual, a utilização equivocada dos termos SAE e PE como sinônimos repercute negativamente na formação da identidade profissional do enfermeiro^(7)^. Por reflexão, entende-se que a supressão do termo SAE da Resolução Cofen 736/2024 diz respeito a uma forma clara, objetiva e pragmática de ressaltar diferenças entre os dois conceitos e que a utilização de ambos como sinônimos é um equívoco que limita a aplicação operacional do PE. Entretanto, do que propõem os historicistas da filosofia da ciência como Thomas Khun e Imre Lakatos, um paradigma ou um programa de pesquisa científica e seus elementos não costumam desaparecer aos primeiros questionamentos^(21)^. Sendo assim, o core conceitual da SAE não deve desaparecer até que seja completamente derrubado pela inexistência de elementos operacionais que permitam uma verificação, uso e testagem; isso se vier a desaparecer um dia.

Partindo da concepção pragmática de que a SAE é um conceito parcialmente maduro^(17)^, ela dispõe de validade conceitual; entretanto, suas propriedades não são plenamente refutáveis. Sendo assim, é relevante para a ciência da disciplina avançar em investigar seus aspectos estruturais e operacionais.

### AVanço dos Elementos Essenciais da SAE no Brasil

O termo “SAE” possivelmente advém de uma proposição da Divisão de Assistência de Enfermagem do Hospital Universitário (HU) da Universidade de São Paulo (USP) que ao elaborar, em 1981, seu marco conceitual e seus objetivos, definiu o objetivo de “estabelecer um sistema adequado de cooperação e colaboração com médicos e outros profissionais, visando a uma assistência integrada”. Para ele, foi dado o nome de “Sistema de Assistência de Enfermagem”. Nesse período, a terminologia passou por modificações, sendo substituída inicialmente de “Sistemática” para “Sistema de Assistência de Enfermagem”^(22)^.

Até então, esse sistema tinha como propósito permitir integrar as ações docente-assistenciais, enquanto estratégia de aprendizado para alunos de graduação, pós-graduação e da própria equipe de enfermagem do HU da USP. No sistema, o “core” direcionador do cuidado profissional de enfermagem é o PE, balizador do saber-fazer dos profissionais da equipe de enfermagem^(22)^. Nesse cenário, foi criado, em 1985, o Grupo de Estudos do Sistema de Assistência de Enfermagem (GESAE). O uso da palavra “sistema” refere-se a um conjunto de elementos inter-relacionados que interagem entre si com um propósito comum, formando um todo organizado, para compartilhamento de dados entre profissionais da equipe multiprofissional^(23,24)^.

Presumivelmente usando a experiência do HU da USP, na década seguinte, o Conselho Regional de Enfermagem de São Paulo publicou a Decisão Coren-SP nº 008/1999 que “normatiza a implementação da SAE nas instituições de saúde no âmbito do Estado de São Paulo”^(14)^. Nesse documento há uma mudança do termo “sistema” para “sistematização”; todavia, não se apresenta explicação para essa alteração terminológica. Essa decisão foi publicada no ano seguinte, com o mesmo texto, pelo Conselho Regional de Enfermagem da Bahia – Decisão Coren-BA nº 007/2000^(15)^.

Nos textos dessas Decisões, alguns pontos na parte dos considerandos permitem inferir que ambas adotam a expressão usada pelas enfermeiras do HU-USP ao usar SAE no masculino (“o SAE”). Também, insere o termo “SAE”, normalizando como uma “atividade privativa do Enfermeiro” que “utiliza método e estratégia de trabalho científico para a identificação das situações de saúde/doença, subsidiando a prescrição e implementação de ações de assistência de enfermagem que possam contribuir para a promoção, prevenção, recuperação e reabilitação em saúde do indivíduo, família e comunidade”. Em seguida traz “a institucionalização do SAE”, aqui novamente no masculino, como a prática de um processo de trabalho adequado às necessidades da comunidade e como modelo assistencial a ser aplicado em todas as áreas de assistência à saúde pelo Enfermeiro”^(14,15)^. Embora em outros trechos exista o uso feminino para palavra SAE, depreende-se uma possível origem no Sistema do HU da USP e de sua acepção organizativa e operacional. Vale destacar que desde a primeira publicação no formato de livro do HU da USP em 1992, é clara a distinção entre o PE e o sistema operacional denominado de SAE.

Além disso, nessas decisões aparecem de modo confuso o uso do termo “PE” como sinônimo de “SAE” ao apresentar em seu artigo primeiro que: “Ao Enfermeiro incumbe: I- privativamente a implantação, planejamento, organização, execução e avaliação do PE, que compreende as seguintes etapas: Consulta de Enfermagem; Histórico e Exame Físico; Diagnóstico de Enfermagem; Prescrição de Enfermagem e Evolução de Enfermagem”^(14,15)^.

É possível observar duas inconsistências, uma no que tange à determinação do PE como atividade privativa do enfermeiro, haja vista que existem etapas do processo que, de fato, são privativas, mas não em sua totalidade. E a outra incongruência passa pela descrição das etapas, pertencentes ao que, internacionalmente, é reconhecido como etapas do PE.

Mais adiante, o texto das decisões estabelece prazos para que ocorra a implementação “do SAE” e traz no seu artigo quinto que: “A implementação do SAE deverá ser registrada formalmente no prontuário do paciente/cliente, devendo ser composta por: Histórico de Enfermagem; Exame Físico; Prescrição da Assistência de Enfermagem; Evolução da Assistência de Enfermagem; Relatório de Enfermagem”^(14,15)^.

Somente dois anos depois o Conselho Federal de Enfermagem publicou a Resolução Cofen nº 272/2002^(16)^, a qual tem seu texto fiel ao das Decisões dos Conselhos Regionais de São Paulo e Bahia, com exceção das datas para acompanhamento do processo de implementação, que não aparece na Resolução. Foi acrescido que cabe a cada regional, em suas respectivas jurisdições, promover encontros, seminários, eventos, para subsidiar técnica e cientificamente os profissionais de Enfermagem na implementação da SAE (no texto da resolução, usa sempre no feminino). Esse movimento avançará para uma indesejável sobreposição conceitual entre SAE e PE que encaminha para a publicação da Resolução Cofen nº 358/2009^(5)^.

Embora a Resolução Cofen nº 358/2009^(5)^ busque apresentar a distinção conceitual, ela não evoca descritivamente uma instrumentalidade particular para a SAE, que seria o instrumento metodológico. Por conta desta condição entendida como aspecto de maturidade parcial^(17)^, o conceito de SAE permanece impreciso e posto à margem do avanço científico da disciplina.

Em 2020, um grupo de pesquisadores de diversas Instituições de Ensino Superior e de Saúde do Brasil criou a Rede de Pesquisa em Processo de Enfermagem (RePPE), com a finalidade de gerar, sintetizar e compartilhar o conhecimento sobre processo e classificações de Enfermagem. Na busca por uma clarificação conceitual da SAE, e em parceria com o Cofen, novo grupo de trabalho foi formado para pensar acerca da Resolução Cofen nº 358/2009^(5)^, culminando com a publicação da Resolução 736/2024^(6)^. Essencialmente, define o PE enquanto método que orienta o pensamento crítico e julgamento clínico do enfermeiro para que esse profissional direcione a equipe de enfermagem no cuidado da pessoa, família, coletividade e grupos especiais, ressaltando a necessidade da utilização de teorias que embasem o método, e detalha as etapas que compõem o PE, a saber: Avaliação de enfermagem; Diagnóstico; Planejamento; Implementação e Evolução^(6)^. Essa última resolução apresenta, para o conceito de PE, elementos estruturais e operacionais bem delimitados, compatíveis com um conceito maduro e passível de verificação de evidências, tanto qualitativas como quantitativas^(20)^.

Entretanto, a literatura dispõe de alguns elementos essenciais distintivos da SAE, visto que parte dos pesquisadores sempre se posicionaram acerca de atributos distintivos dos conceitos. Publicações^(17,25)^ atribuem propriedades intimamente relacionadas com a gestão dos serviços de enfermagem, de modo a organizar o trabalho a partir dos seus três pilares (método, pessoal, instrumentos), que podem ser utilizados para auxiliar na compreensão do nível organizacional dos serviços de enfermagem.

Duas perspectivas podem facilitar os esforços de amadurecimento do conhecimento teórico-conceitual e operacional de SAE fora dos limites estabelecidos claramente no PE: o de *Professional Practice Models* (PPM)^(26)^ e o de um modelo ou de uma ferramenta de gestão operando no âmbito de um raciocínio clínico^(27)^.

Um Modelo de Prática Profissional (PPM, acrônimo em inglês) é uma estrutura conceitual que oferece para a enfermagem fundamentos para um cuidado seguro, centrado no paciente e de alta qualidade, indicando para além dos valores, as estruturas e processos que apoiam os enfermeiros no controle da sua prática e do ambiente de cuidados^(26,28)^. Em seus fundamentos teóricos o PPM costuma englobar: a governança compartilhada como conceito central, valores centrais como base, aplicação de teorias de enfermagem que explicam a natureza do cuidado e da performance profissional, e teorias organizacionais, ou ainda modelos^(28)^.

Na concepção de um raciocínio clínico, de certo modo ampliado^(27)^, o conceito de SAE mantém seu núcleo de gestão. Entretanto esta gestão é mais bem delimitada em seus componentes operacionais e estruturais definidos no tópico seguinte.

### A SAE Entre Modelos de Prática Profissional e O Raciocínio Clínico: uma Análise Operacional e Estrutural

Estudo de Análise de Conceito sobre SAE^(17)^ conclui ser este um conceito parcialmente maduro, pautado por múltiplas definições e que se operacionaliza a partir de outros conceitos. Nessa perspectiva vale destacar que o mesmo estudo^(17)^ identificou os primeiros registros sobre o uso linguístico dos termos “sistematização” e “sistematizar”, nos anos de 1960, demonstrando uma característica de organização da prática profissional em prol de adequadas condições para o trabalho clínico do enfermeiro, para além do que já foi apresentado nessa reflexão teórica com relação a uma hipótese da origem do termo “SAE” advindo de uma proposição da Divisão de Assistência de Enfermagem do HU da USP com modificações no seu percurso quando das publicações dos Conselhos Regionais de Enfermagem e a *posteriori* do Conselho Federal.

Editorial, escrito por uma pesquisadora com livre-docência na área da Administração em Enfermagem, tratando da administração da assistência de enfermagem, atualmente denominado de Gestão em Enfermagem, pontua que ela estaria voltada, principalmente, para atender às necessidades de selecionar e adequar métodos de organização do trabalho, o que a autora destaca entre parênteses como SAE^(29)^.

Partindo desse fio condutor, um estudo recente^(25)^ considera que a SAE não se configura como objeto epistemológico com conexão convergente ao cuidado direto ao paciente, mas que é um conceito que reúne propriedades que lhe asseguram um campo de conhecimento relacionado à gestão e/ou à administração dos serviços de enfermagem, enquanto um modelo ou até mesmo uma ferramenta para gestão do cuidado^(7)^. Sob essa perspectiva, a SAE abrange dimensões que transcendem o cuidado direto ao paciente e possibilita análise e reflexão quanto à eficiência e eficácia das ações desenvolvidas pela equipe de enfermagem, com o intuito de contribuir para tomada de decisão na gestão e políticas dos serviços, visando a excelência do cuidado.

Reconhecendo que a SAE é um construto genuinamente brasileiro, ainda que sob o risco de vir a ser proscrito, importa destacar que, à luz da filosofia da ciência – especialmente da noção de “programas de pesquisa científica” proposta por Imre Lakatos^(21)^, teorias e modelos não são abandonados diante das primeiras dificuldades ou críticas. Ao contrário, os grupos que aplicam tais estruturas como explicações tendem a investir em desenvolvimento e refinamento, ajustando-as frente às questões levantadas. Abandonar a concepção de uma SAE neste momento equivaleria a ceder à lógica de um descarte precoce, em detrimento do amadurecimento de um modelo teóricoempírico com potencial de orientar e fortalecer a gestão da prática profissional e científica da Enfermagem.

Como já mencionado, a existência de construtos potencialmente correlatos à SAE em outros contextos internacionais, a exemplo do PPM^(17)^, reitera a relevância em considerar as possibilidades de um modelo singular do Brasil. Em certa medida, um PPM dispõe de atributos convergentes aos fundamentos do que seria a SAE, apresentando-se como uma lente ampliada e estruturante, capaz de ressignificar a SAE, especialmente, na proposição de uma abordagem sistêmica, integradora de valores profissionais, processos assistenciais, relações interprofissionais, mecanismos de reconhecimento e modelos de governança.

A estrutura dos PPM é composta por componentes essenciais que definem sua organização e funcionamento, tais como: valores profissionais^(26)^, uma base teórica e conceitual que sustenta o modelo^(28)^, relações profissionais colaborativas, organização da assistência ao paciente^(26,28)^, abordagem de gestão participativa^(26)^, liderança em todos os níveis da prática^(28)^ e mecanismos de reconhecimento e valorização do enfermeiro^(26,28)^.

Ainda mais, os PPM configuram-se como dispositivos epistemológicos que conferem à Enfermagem um espaço autônomo, deliberativo e reflexivo, sustentado por valores fundantes como dignidade, responsabilidade e compromisso ético-estético para um cuidado de excelência^(26,28)^. Ao instituírem uma arquitetura conceitual que articula saber, fazer e ser profissional, esses modelos não apenas orientam a prática assistencial, mas também ressignificam os processos de tomada de decisão na gestão e políticas dos serviços de enfermagem, contribuindo para a qualificação dos ambientes de cuidado e a ampliação dos horizontes da disciplina^(7)^.

A SAE, conforme delineada na literatura, fundamenta-se na organização do trabalho da equipe de enfermagem^(30)^, estruturalmente articulada em três pilares fundamentais – método, pessoal e instrumentos^(25,31)^. Esses pilares visam instituir uma dinâmica laboral orientada por um sistema próprio, centrado na gestão do cuidado^(10)^, com o propósito de estruturar e qualificar o *saber-fazer* da equipe de enfermagem no cotidiano assistencial.

Neste contexto, torna-se imperativo que as lideranças da Enfermagem reconheçam que a partilha e a consolidação de crenças, valores, saberes e práticas não são apenas estratégias organizacionais, mas fundamentos ontológicos e epistemológicos para a (re)construção da identidade profissional^(7)^. Por analogia aos PPM, a SAE aponta, portanto, para a necessidade de um pensamento crítico, comprometido com a produção de sentidos que transcendam a mera operacionalização técnica e avancem em direção à consolidação da Enfermagem como ciência aplicada e prática social complexa.

Compreendida sob a ótica de um PPM, a SAE apresenta múltiplos atributos: atua como modelo assistencial, método de trabalho para organização de processos, dispositivo organizacional, instrumento de gestão e via para a efetivação de um cuidado sistematizado, qualificado e responsivo às necessidades em saúde^(30)^.

Assim, sua finalidade epistemológica e operacional reside em oferecer diretrizes para a concretização das ações de cuidado, orientadas pelo PE nos distintos cenários assistenciais da gerência do cuidado. Trata-se, portanto, de uma modelo ou ferramenta estruturante disponível para o *saber-fazer* profissional, útil para articular teoria e prática, conhecimento e ação, individual e coletivo, sendo um marco identitário da Enfermagem brasileira.

Em sua estrutura, concordamos com os autores^(25,31)^ ao reconhecer que o pilar “método” pode ser compreendido à luz dos valores profissionais da Enfermagem, ancorados numa epistemologia própria – especialmente nas Teorias de Enfermagem e no PE. Este, por sua vez, constitui-se como eixo articulador do pensamento crítico e do julgamento clínico do enfermeiro, orientando a equipe de enfermagem na prestação do cuidado à pessoa, família, coletividade e grupos em situações específicas de vulnerabilidade. No âmbito institucional, esse pilar também se relaciona com a forma como o cuidado é organizado metodologicamente no serviço, considerando a missão, a filosofia e os objetivos da instituição de saúde, bem como a definição de modelos assistenciais que orientam a prática da enfermagem. Assim, o método materializa-se na adoção de referenciais teóricos, na operacionalização das etapas do PE e na forma como o cuidado é planejado, executado e avaliado no cotidiano dos serviços.

O pilar “pessoal” compreende aspectos relacionados ao dimensionamento da força de trabalho, à gestão de pessoas e à formação e desenvolvimento de recursos humanos, fundamentais para sustentar uma prática profissional qualificada e alinhada às demandas assistenciais^(25,31)^. Nessa dimensão incluem-se elementos como a disponibilidade e o adequado dimensionamento da equipe de enfermagem, as estratégias institucionais de capacitação e educação permanente, a sensibilização da equipe para a importância da SAE, bem como o desenvolvimento de competências clínicas e gerenciais necessárias à implementação do PE. Também se inserem nesse pilar aspectos relacionados ao interesse institucional e ao compromisso da gestão em apoiar a implementação da SAE, garantindo condições organizacionais e estruturais para sua efetivação.

Por fim, o pilar “instrumentos” estrutura-se a partir de um conjunto de dispositivos normativos e operacionais que dão suporte à prática do cuidado, tais como: manuais de enfermagem, regimentos internos do serviço, protocolos operacionais padrão (POP), protocolos assistenciais, *bundles* clínicos, escalas de avaliação e formulários próprios da enfermagem. Esses instrumentos não apenas operacionalizam o cuidado, como também contribuem para sua padronização, segurança e qualidade, em consonância com os princípios da SAE e respeitando os preceitos da política de segurança do paciente. Incluem-se ainda nessa dimensão os impressos e sistemas de registro utilizados pela equipe de enfermagem para documentar as etapas do PE – como formulários de coleta de dados, registros de diagnósticos, planejamento, prescrições e evolução de enfermagem – além de instrumentos de avaliação clínica que considerem as características da clientela atendida e o perfil assistencial das unidades.

Diante do exposto, é possível afirmar que os pilares estruturantes da SAE guardam estreita correspondência com os componentes essenciais dos Modelos de Prática Profissional descritos na literatura internacional. O pilar “método” incorpora o PE, elemento central da prática clínica, e os referenciais teóricos da disciplina capazes de fornecer descrições, explicações, predições e prescrições sobre fenômenos e eventos profissionais. O pilar “pessoal”, ao abranger a gestão de pessoas, dimensionamento e qualificação da equipe, converge com os componentes relativos à liderança, desenvolvimento profissional e ambiente de prática. Já o pilar “instrumentos”, ao reunir protocolos, regimentos, formulários e outros dispositivos normativos, dialoga com os elementos dos PPM que estruturam a governança, padronização da assistência e suporte à tomada de decisão clínica. Assim, compreende-se a SAE não apenas como um arranjo técnico-operacional, mas como um sistema integrado de fundamentos epistemológicos, organizacionais e práticos, capaz de potencializar tanto o raciocínio clínico quanto a gestão do cuidado em múltiplas dimensões.

Essas correspondências evidenciam que a SAE, enquanto constructo genuinamente brasileiro, pode e deve ser compreendida como uma expressão situada e robusta de um Modelo de Prática Profissional, reafirmando sua relevância como fundamento organizativo, epistemológico e ético do cuidado em Enfermagem. Dessa forma, amplia-se a extensão conceitual e modelar da SAE para além de uma ferramenta organizacional, oferecendo potencial como estrutura representativa da integração de conhecimento e prática.

O enfermeiro, enquanto gestor do cuidado, deve pensar a organização de todo o serviço e direcionar o planejamento dos cuidados com base no PE e no raciocínio clínico – compreendido como processo dinâmico que articula cognição, metacognição e tomada de decisão^(27)^. Tal pensamento de natureza sistêmica, em tese, promove a excelência do cuidado capaz de extrapolar o diagnóstico para abarcar decisões terapêuticas, monitoramento e avaliação contínua deste.

Em perspectiva sistêmica, a SAE fortalece-se como dispositivo de gestão do cuidado num sentido mais amplo e estratégico, ligado à tomada de decisões macropolíticas, de planejamento, avaliação de resultados e políticas institucionais^(8,10)^, estruturando a prática com base na autonomia profissional, na competência técnica e no compromisso com os sujeitos do cuidado. Essa compreensão exige a qualificação permanente da equipe, o alinhamento às diretrizes legais e o reconhecimento da SAE como expressão de um modelo de prática fundamentado em valores profissionais e no escopo da ciência da Enfermagem. A articulação entre SAE, raciocínio clínico e os fundamentos dos PPM oferece, portanto, um caminho promissor para o amadurecimento do conceito de SAE no Brasil, contribuindo para a coerência teórico-prática do cuidado, a gestão qualificada dos serviços e o fortalecimento identitário da Enfermagem enquanto disciplina e campo profissional.

Embora as reflexões deste estudo possam avançar o conhecimento, existe uma limitação fundamental. O estudo tem limites em fornecer aspectos instrumentais específicos para aplicação distinta da SAE, o que é essencial para garantir que não se produzam novas sobreposições conceituais ou que se esvaziem avanços obtidos da clarificação da maturidade conceitual do PE para a prática clínica. Entretanto, este limite depende de superações contínuas em futuras pesquisas que possam utilizar evidências trazidas e transponíveis dos PPM para a realidade de implantação de modelos de SAE no Brasil.

## CONCLUSÃO

À guisa das considerações finais, a SAE, apesar de sua trajetória marcada por ambiguidades conceituais e disputas semânticas, permanece como um constructo importante para a organização da prática profissional da Enfermagem no Brasil. Seu reconhecimento como análogo a um Modelo de Prática Profissional, alinhado aos pilares “método”, “pessoal” e “instrumentos”, permite reinterpretá-la não como sinônimo de PE, mas como um modelo de gestão do cuidado. A correlação entre esses pilares e os componentes essenciais dos PPM reforça a potencialidade da SAE em estruturar o trabalho da equipe de enfermagem de forma crítica, reflexiva e integrada, promovendo não apenas a qualidade da assistência, mas também o fortalecimento identitário da Enfermagem.

Sua permanência no campo disciplinar não depende apenas de sua tradição normativa ou de seu enraizamento cultural, mas da capacidade de ser reconfigurada à luz de fundamentos epistemológicos sólidos, da prática baseada em evidências e das demandas contemporâneas dos serviços de saúde. Reafirmar a SAE como um modelo implica reposicioná-la como capaz de sustentar a dimensão macropolítica de planejamento, de modo a orientar uma abordagem de gestão participativa e marcada pela liderança do enfermeiro de modo a contribuir para a excelência do cuidado em Enfermagem no Brasil na articulação em rede e nas políticas do SUS.

Conclui-se reafirmando o compromisso ético desta análise com a autoria intelectual das fontes. A interpretação das produções científicas e normativas preservou a integridade das ideias originais, assegurando que as sínteses elaboradas não distorcessem o pensamento dos autores citados.

## Data Availability

Todo o conjunto de dados que dá suporte aos resultados deste estudo foi publicado no próprio artigo.

# Translation: Sistematización de los cuidados de enfermería como modelo de práctica profesional de la enfermería en Brasil: uma propuesta crítico-epistemológica

**DOI:** 10.1590/1980-220X-REEUSP-2025-0551es

**Authors:** Rudval Souza da Silva, Marcos Antônio Gomes Brandão, Alba Lúcia Bottura Leite de Barros, Marcia Regina Cubas, Jackeline Felix de Souza, Ana Raquel Lima Peralva de Almeida, Laura Emmanuela Lima Costa

**Affiliations:** 1 Universidade do Estado da Bahia, Programa de Pós-graduação em Ciências do Cuidar e Saúde, Senhor do Bonfim, BA, Brasil.; 2 Universidade Federal do Rio de Janeiro, Escola de Enfermagem Anna Nery, Rio de Janeiro, RJ, Brasil.; 3 Universidade Federal de São Paulo, Escola Paulista de Enfermagem, São Paulo, SP, Brasil.; 4 Pontifícia Universidade Católica do Paraná, Programa de Pós-Graduação em Tecnologia em Saúde, Curitiba, PR, Brasil.; 5 Universidade Federal da Bahia, Programa de Pós-graduação em Enfermagem e Saúde, Salvador, BA, Brasil.; 6 Universidade do Estado da Bahia, Jacobina, BA, Brasil.

**Keywords:** Atención de Enfermería, Práctica Profesional, Proceso de Enfermería, Organización y Administración

## Abstract

**Objetivo:**

Analizar la historiografía del concepto de Sistematización de Cuidados de Enfermería (SAE) como modelo de práctica profesional de enfermería en Brasil, avanzando en la comprensión de sus límites conceptuales y operativos.

**Método:**

Ensayo teórico-reflexivo, basado en investigación documental. Se examinaron actos normativos en un intento de analizar el concepto de SAE, además de libros de referencia y artículos indexados, que conforman la historiografía de la SAE en Brasil y sus interfaces con el Proceso de Enfermería. El análisis se realizó mediante lectura analítica y síntesis narrativa.

**Resultados:**

Los pilares estructurales del concepto de SAE, “método”, “personal” e “instrumentos”, guardan una estrecha correspondencia con los componentes esenciales de los Modelos de Práctica Profesional descritos en la literatura internacional.

**Conclusión:**

Se concluye que la SAE, a pesar de su trayectoria marcada por ambigüedades conceptuales, sigue siendo un constructo significativo en la organización de la práctica profesional de la enfermería brasileña. Se sitúa como un Modelo de Práctica Profesional; alineado con sus pilares, permite reinterpretarlo no como sinónimo de Proceso de Enfermería, sino como un modelo ampliado de gestión de la atención en una dimensión de la macropolítica de planificación, articulación en red y políticas del SUS.

## INTRODUCCIÓN

La enfermería, en un escenario histórico y global, se configura como una disciplina científica y un campo profesional que fundamenta su práctica en la promoción de la salud, la seguridad para la continuidad de la atención, así como en la gestión y el liderazgo de las organizaciones y los sistemas de salud^(1)^. De esta manera, alcanza un estado de disciplina consolidada, con un corpus de conocimientos propios apoyado en la ciencia^(2)^. Ante esto, para alcanzar sus objetivos, los profesionales del área se esfuerzan por cuidar, a partir de una práctica basada en la evidencia, considerando las respuestas humanas y las necesidades básicas —afectadas o no— que sustentan la vida, mediante un método propio conocido mundialmente como Proceso de Enfermería (PE), una adaptación del método científico a su campo disciplinario^(1,2)^.

A partir de las contribuciones de Wanda de Aguiar Horta, el PE llega al panorama de la enfermería brasileña en los últimos años de la década de 1970. Su perspectiva de la urgencia del PE ya se puede observar en el prefacio de la obra “Proceso de Enfermería”, que ganó notoriedad en el país al afirmar que “la autonomía profesional solo se alcanzará en el momento en que toda la clase comience a utilizar la metodología científica en sus acciones, lo cual solo se logrará mediante la aplicación sistemática del PE”^(3)^.

Desde entonces, diversas publicaciones de resultados de investigaciones han demostrado la consolidación del PE en el panorama brasileño. Concomitantemente a este avance, se ha observado el uso erróneo de la sinonimia entre los términos “PE” y “sistematización de la atención de enfermería – SAE”.

En una búsqueda asistémica en la Revista Brasileña de Enfermería, la primera revista del área en Brasil, que data de 1932, se pudo observar que en la década de 1980 se utilizaba el término “metodología de la atención de enfermería – MAE”; en la década siguiente destaca el uso del término “PE” y a partir del año 2000 surge, en las normativas del Sistema Cofen/Consejos Regionales, el uso del término “SAE”, que a veces se utiliza como un sistema de gestión y otras como sinónimo de PE. En este estudio, entendemos que se trata de términos distintos y que requieren una comprensión conceptual para su aplicabilidad en el ámbito teórico-práctico.

Esta problemática conceptual ha generado, hasta el momento de este estudio, la publicación de tres Resoluciones por parte del Consejo Federal de Enfermería. Esto nos permite considerar que es necesario reflexionar al respecto y, para ello, es importante conocer la historiografía de estos conceptos, en especial sobre el concepto “SAE” acuñado por enfermeras brasileñas y difundido por todas partes, en este Brasil continental y objeto de esta reflexión teórica.

A finales de la primera década del siglo XXI, se llevó a cabo un estudio que buscó identificar las corrientes de pensamiento sobre la SAE, identificando tres concepciones de pensamiento. Una primera que entiende que existe una diferencia conceptual entre los términos SAE, MAE y PE. Un segundo subgrupo que considera equivalentes los términos PE y MAE y un tercero que entiende los tres términos como sinónimos^(4)^. Es importante destacar que la Resolución Cofen n.º 358/2009^(5)^, derogada con la publicación de la Resolución Cofen n.º 736/2024^(6)^, y los autores de este ensayo reflexivo consideran que se trata de conceptos distintos.

Dicho esto, entendemos que es relevante analizar la historiografía del concepto SAE, con una mirada orientada hacia el Modelo de Práctica Profesional. Además, se busca comprender el contexto histórico actual y si esta ramificación epistemológica tiene cabida en la enfermería brasileña en el ámbito de la gestión de la atención, con miras a apoyar su uso adecuado por parte de los profesionales del campo de la enfermería y la salud, en la medida en que el pensamiento de gestión y organización sobre la SAE y su alineación con el PE sustente fundamentos teóricometodológicos para la gestión de la atención en enfermería. Asimismo, se espera avanzar ante la necesidad de aclarar los conceptos en pro de una posible comprensión. Dado que la SAE es una expresión eminentemente utilizada en las publicaciones de Brasil, resulta esencial que los investigadores brasileños agoten las cuestiones conceptuales y operativas sobre la temática, avanzando en su comprensión.

Tras esta breve contextualización y problematización, nos corresponde cuestionar, y con ello ampliar las reflexiones: cuando usamos la expresión SAE, ¿cuál es nuestro entendimiento? ¿Cómo se originó esta expresión? ¿Es un concepto que debe abandonarse? ¿Ya no logra explicar los fenómenos observados? ¿O será que, al dejarlo en desuso, no estamos deslegitimando una subjetividad cultural de una expresión tan arraigada en las producciones científicas y en la práctica de la enfermería brasileña?

En un intento por crear las condiciones para tal análisis y presentar posibles reflexiones epistemológicas a estas preguntas, se considera esencial presentar una historiografía sobre el concepto de SAE en Brasil, analizando los elementos que lo sustentan con el fin de reforzar las distinciones y reiterar el concepto de PE como instrumento metodológico para pensar, planificar, ejecutar y evaluar la atención profesional que refleja la práctica del equipo de enfermería. Sin embargo, el análisis se justifica aún más en las posibilidades de articular el PE con un Modelo de Práctica Profesional de la enfermería brasileña que es la SAE, como modelo para la gestión de la atención de enfermería que trasciende el ámbito de la atención directa, permitiendo evaluar la eficiencia y la eficacia de las acciones desarrolladas, además de respaldar decisiones gerenciales y políticas centradas en la cualificación y la excelencia de la atención prestada ^(7)^.

Cabe destacar las diferencias conceptuales entre gestión^(8)^ y administración^(9)^ de la atención de enfermería, como constructo nacional, resultantes de la constitución epistemológica de un corpus literario nacional sobre el proceso de trabajo de enfermería, con miras a la integración de estas dimensiones en la actuación del enfermero. La gestión de la atención se constituye a partir de una actuación macropolítica del enfermero en la Red de Atención de Salud del Sistema Único de Salud (SUS), orientando la movilización de recursos y equipos. La SAE organiza y califica el proceso de trabajo del equipo de enfermería, con el fin de garantizar la integralidad de la atención y hacer efectivo el derecho a la salud. Por su parte, la gestión de la atención corresponde a la dimensión micropolítica de la práctica del enfermero, centrada en la ejecución y coordinación del PE en el día a día de los servicios de salud. Se fundamenta en el trabajo en equipo interdisciplinario, asegurando la calidad y la continuidad de la atención de enfermería, no actuando de manera aislada, sino con profesionales del equipo de enfermería, así como con profesionales de otros equipos^(10)^.

Así, el presente estudio teórico-reflexivo tiene como objetivo analizar la historiografía del concepto SAE como Modelo de Práctica Profesional de Enfermería en Brasil, avanzando en la comprensión de sus límites conceptuales y operativos.

### Historiografía de la SAE en Brasil

Mediante una investigación documental, fue posible identificar la primera publicación brasileña de mayor difusión que, en Brasil, pudo haber expandido una concepción de sistematización de la práctica profesional: el libro “Proceso de Enfermería” de Horta^(3)^. Se señala como la de mayor difusión, ya que Wanda de Aguiar Horta ya había publicado anteriormente artículos científicos sobre las funciones de la enfermería y los desafíos de la organización de la práctica mediante la determinación del diagnóstico de enfermería, la elaboración y ejecución del plan de cuidados^(11)^; la propuesta de la metodología del PE^(12)^; el desarrollo de teorías y terminologías científicas de enfermería y de un plan de atención^(13)^, entre otros.

Posteriormente, se publicaron documentos normativos de los consejos de la profesión, entre los que destacan los del Consejo Regional de Enfermería de São Paulo^(14)^ y de Bahía^(15)^, que dieron origen, posteriormente, a la Resolución Cofen n.º 272/2002^(16)^, derogada al igual que la Resolución Cofen n.º 358/2009^(5)^, y cuyo marco regulatorio actual es la Resolución Cofen n.º 736/2024(6). Cabe destacar que en este último documento no se menciona el concepto SAE.

A lo largo de la trayectoria histórica de la enfermería, se observa que, a partir de la década de 1960, se intensificó en Brasil el interés por organizar y fundamentar la práctica profesional sobre bases teóricas y metodológicas, en consonancia con el movimiento internacional de la PE. Sin embargo, no fue hasta la década de 1980 que la expresión “SAE” comenzó a aparecer en publicaciones científicas, de manera más efectiva en la literatura brasileña, a menudo utilizada como sinónimo de PE^(17)^. Esta migración semántico-conceptual condujo a la tendencia de uso y superposición conceptual abordada en trabajos que tenían como objetivo aclarar las tenues líneas de diferenciación conceptual de los términos^(4,17)^.

Una vez más, el carácter normativo tiende a buscar la síntesis de los debates académicos. De este modo, el marco regulatorio, expedido por el Cofen mediante la Resolución n.º 358/2009, incluye en sus considerandos una definición de la SAE como un recurso que “organiza el trabajo profesional en cuanto a método, personal e instrumentos, haciendo posible la puesta en práctica del PE”^(5)^.

Esta misma definición se encuentra en un libro^(18)^ publicado en 2006 cuyo título es Teoría y Método en la Asistencia de Enfermería y ya provocaba esta reflexión relacionada con tal embrollo conceptual, asumiendo que se trata de conceptos distintos. Teniendo en cuenta que la Resolución n.º 358/2009 no incluye las referencias adoptadas y, considerando que la definición de SAE contenida en dicha resolución es *ipsis litteris* a lo que define como SAE la autora^(18)^, es posible inferir que pudo haber sido una referencia adoptada por el grupo que elaboró la mencionada resolución de 2009.

Esta Resolución, en cierto modo, divide a la comunidad académica en dos interpretaciones centrales. La primera, de que la SAE tendría entre sus atributos exclusivos elementos de gestión y administración de enfermería^(17)^; y la segunda, de que se trata de un uso inapropiado del PE, siendo este último el que debe emplearse^(19)^.

En 2024, se publica la Resolución Cofen n.º 736/2024 omitiendo la mención a la SAE, y abordando exclusivamente el PE, que se define como “un método que orienta el pensamiento crítico y el juicio clínico del enfermero, dirigiendo al equipo de enfermería hacia el cuidado de la persona, la familia, la colectividad y los grupos especiales”^(6)^. A partir de la mencionada Resolución, la comunidad académica parece reconocer claramente la conceptualización distintiva entre SAE y PE en términos teórico-conceptuales y operativos, y otorga protagonismo al PE, lo que desde la perspectiva pragmática conceptual se consideró el “concepto maduro”^(17,20)^.

En el estado actual del conocimiento, el uso erróneo de los términos SAE y PE como sinónimos repercute negativamente en la formación de la identidad profesional del enfermero^(7)^. Por reflexión, se entiende que la supresión del término SAE de la Resolución Cofen 736/2024 se refiere a una forma clara, objetiva y pragmática de resaltar las diferencias entre los dos conceptos y que el uso de ambos como sinónimos es un equívoco que limita la aplicación operativa del PE. Sin embargo, según lo que proponen los historicistas de la filosofía de la ciencia como Thomas Kuhn e Imre Lakatos, un paradigma o un programa de investigación científica y sus elementos no suelen desaparecer ante los primeros cuestionamientos^(21)^. Por lo tanto, el núcleo conceptual de la SAE no debería desaparecer hasta que sea completamente derribado por la inexistencia de elementos operativos que permitan su verificación, uso y prueba; si es que llega a desaparecer algún día.

Partiendo de la concepción pragmática de que la SAE es un concepto parcialmente maduro^(17)^, esta cuenta con validez conceptual; sin embargo, sus propiedades no son plenamente refutables. Por lo tanto, es relevante para la ciencia de la disciplina avanzar en la investigación de sus aspectos estructurales y operativos.

### Avance de los Elementos Esenciales de la SAE en Brasil

El término “SAE” proviene posiblemente de una propuesta de la División de Asistencia de Enfermería del Hospital Universitario (HU) de la Universidad de São Paulo (USP) que, al elaborar, en 1981, su marco conceptual y sus objetivos, definió el objetivo de “establecer un sistema adecuado de cooperación y colaboración con médicos y otros profesionales, con miras a una atención integrada”. A este se le dio el nombre de “Sistema de Asistencia de Enfermería”. En ese período, la terminología sufrió modificaciones, sustituyéndose inicialmente “Sistemática” por “Sistema de Asistencia de Enfermería”^(22)^.

Hasta entonces, este sistema tenía como propósito permitir integrar las acciones docentes y asistenciales, como estrategia de aprendizaje para estudiantes de pregrado, posgrado y el propio equipo de enfermería del HU de la USP. En el sistema, el “núcleo” que orienta la atención profesional de enfermería es el PE, que marca el saber hacer de los profesionales del equipo de enfermería^(22)^. En este contexto, se creó en 1985 el Grupo de Estudios del Sistema de Asistencia de Enfermería (GESAE). El uso de la palabra “sistema” se refiere a un conjunto de elementos interrelacionados que interactúan entre sí con un propósito común, formando un todo organizado, para el intercambio de datos entre los profesionales del equipo multiprofesional^(23,24)^.

Presumiblemente basándose en la experiencia del HU de la USP, en la década siguiente, el Consejo Regional de Enfermería de SP publicó la Decisión Coren-SP n.º 008/1999 que “normatiza la implementación de la SAE en las instituciones de salud en el ámbito del Estado de SP”^(14)^. En este documento se observa un cambio del término “sistema” a “sistematización”; sin embargo, no se ofrece explicación alguna para este cambio terminológico. Esta decisión fue publicada al año siguiente, con el mismo texto, por el Consejo Regional de Enfermería de Bahía: Decisión Coren-BA n.º 007/2000^(15)^.

En los textos de estas Decisiones, algunos puntos de los considerandos permiten inferir que ambas adoptan la expresión utilizada por las enfermeras del HU-USP al referirse al SAE en masculino (“el SAE”). Además, se introduce el término “SAE”, normalizándolo como una “actividad exclusiva del enfermero” que “utiliza métodos y estrategias de trabajo científico para la identificación de situaciones de salud/enfermedad, subsidiando la prescripción e implementación de acciones de asistencia de enfermería que puedan contribuir a la promoción, prevención, recuperación y rehabilitación en salud del individuo, la familia y la comunidad”. A continuación, presenta “la institucionalización del SAE”, aquí nuevamente en masculino, como la práctica de un proceso de trabajo adecuado a las necesidades de la comunidad y como modelo asistencial que debe aplicar el enfermero en todas las áreas de la atención de la salud”^(14,15)^. Aunque en otros pasajes se utiliza la forma femenina para la palabra SAE, se deduce un posible origen en el Sistema del HU de la USP y en su acepción organizativa y operativa. Cabe destacar que, desde la primera publicación en formato de libro del HU de la USP en 1992, es clara la distinción entre el PE y el sistema operativo denominado SAE.

Además, en estas decisiones aparece de manera confusa el uso del término “PE” como sinónimo de “SAE” al presentar en su primer artículo que: “Al enfermero le incumbe: I- de manera exclusiva la implementación, planificación, organización, ejecución y evaluación del PE, que comprende las siguientes etapas: Consulta de enfermería; Historia clínica y examen físico; Diagnóstico de enfermería; Prescripción de enfermería y Evolución de enfermería”^(14,15)^.

Se pueden observar dos inconsistencias, una en lo que respecta a la determinación del PE como actividad exclusiva del enfermero, dado que existen etapas del proceso que, de hecho, son exclusivas, pero no en su totalidad. Y la otra incongruencia se refiere a la descripción de las etapas, que pertenecen a lo que, internacionalmente, se reconoce como etapas del PE.

Más adelante, el texto de las decisiones establece plazos para que se lleve a cabo la implementación “del SAE” y establece en su artículo quinto que: “La implementación del SAE deberá registrarse formalmente en el expediente del paciente/cliente, y deberá estar compuesta por: Historial de Enfermería; Examen físico; Prescripción de la atención de enfermería; Evolución de la atención de enfermería; Informe de enfermería”^(14,15)^.

Solo dos años después, el Consejo Federal de Enfermería publicó la Resolución Cofen n.º 272/2002^(16)^, cuyo texto es fiel al de las Decisiones de los Consejos Regionales de São Paulo y Bahía, con excepción de las fechas para el seguimiento del proceso de implementación, que no aparecen en la Resolución. Se añadió que corresponde a cada consejo regional, en sus respectivas jurisdicciones, promover encuentros, seminarios y eventos para apoyar técnica y científicamente a los profesionales de enfermería en la implementación de la SAE (en el texto de la resolución, siempre se utiliza el género femenino). Este movimiento avanzará hacia una superposición conceptual indeseable entre la SAE y la PE que conduce a la publicación de la Resolución Cofen n.º 358/2009^(5)^.

Aunque la Resolución Cofen n.º 358/2009^(5)^ busca presentar la distinción conceptual, no evoca de manera descriptiva una instrumentalidad particular para la SAE, que sería el instrumento metodológico. Debido a esta condición, entendida como un aspecto de madurez parcial^(17)^, el concepto de SAE permanece impreciso y al margen del avance científico de la disciplina.

En 2020, un grupo de investigadores de diversas instituciones de educación superior y de salud de Brasil creó la Red de Investigación en Procesos de Enfermería (RePPE), con el fin de generar, sintetizar y compartir el conocimiento sobre los procesos y las clasificaciones de enfermería. En la búsqueda de una clarificación conceptual de la SAE, y en colaboración con el Cofen, se formó un nuevo grupo de trabajo para reflexionar sobre la Resolución Cofen n.º 358/2009^(5)^, lo que culminó con la publicación de la Resolución 736/2024^(6)^. Esencialmente, define el PE como un método que orienta el pensamiento crítico y el juicio clínico del enfermero para que este profesional dirija al equipo de enfermería en el cuidado de la persona, la familia, la colectividad y los grupos especiales, resaltando la necesidad de utilizar teorías que fundamenten el método, y detalla las etapas que componen el PE, a saber: Evaluación de enfermería; Diagnóstico; Planificación; Implementación y Evolución^(6)^. Esta última resolución presenta, para el concepto de PE, elementos estructurales y operativos bien delimitados, compatibles con un concepto maduro y susceptible de verificación de evidencia, tanto cualitativa como cuantitativa^(20)^.

Sin embargo, la literatura cuenta con algunos elementos esenciales distintivos de la SAE, ya que parte de los investigadores siempre se han posicionado respecto a los atributos distintivos de los conceptos. Las publicaciones^(17,25)^ atribuyen propiedades íntimamente relacionadas con la gestión de los servicios de enfermería, con el fin de organizar el trabajo a partir de sus tres pilares (método, personal, instrumentos), que pueden utilizarse para ayudar a comprender el nivel organizacional de los servicios de enfermería.

Dos perspectivas pueden facilitar los esfuerzos de maduración del conocimiento teórico —conceptual y operativa— de la SAE fuera de los límites establecidos claramente en el PE: la de los *Professional Practice Models* (PPM)^(26)^ y la de un modelo o una herramienta de gestión que opera en el ámbito del razonamiento clínico^(27)^.

Un Modelo de Práctica Profesional (PPM, usando la sigla en inglés) es una estructura conceptual que ofrece a la enfermería los fundamentos para una atención segura, centrada en el paciente y de alta calidad, indicando, más allá de los valores, las estructuras y los procesos que apoyan a los enfermeros en el control de su práctica y del entorno de atención^(26,28)^. En sus fundamentos teóricos, el PPM suele abarcar: la gobernanza compartida como concepto central, valores centrales como base, la aplicación de teorías de enfermería que explican la naturaleza de la atención y el desempeño profesional, y teorías organizacionales, o incluso modelos^(28)^.

En la concepción de un razonamiento clínico, en cierto modo ampliado^(27)^, el concepto de SAE mantiene su núcleo de gestión. Sin embargo, esta gestión está mejor delimitada en sus componentes operativos y estructurales, definidos en el siguiente apartado.

### La SAE Entre los Modelos de Práctica Profesional y el Razonamiento Clínico: un Análisis Operacional y Estructural

Un estudio de análisis conceptual sobre la SAE^(17)^ concluye que se trata de un concepto parcialmente maduro, regido por múltiples definiciones y que se operacionaliza a partir de otros conceptos. Desde esta perspectiva, cabe destacar que el mismo estudio^(17)^ identificó los primeros registros sobre el uso lingüístico de los términos “sistematización” y “sistematizar” en la década de 1960, lo que demuestra una característica de organización de la práctica profesional en pro de condiciones adecuadas para el trabajo clínico del enfermero, más allá de lo que ya se ha presentado en esta reflexión teórica con respecto a una hipótesis sobre el origen del término “SAE”, derivado de una propuesta de la División de Asistencia de Enfermería del HU de la USP, con modificaciones en su trayectoria al momento de las publicaciones de los Consejos Regionales de Enfermería y, a posteriori, del Consejo Federal.

Un editorial, escrito por una investigadora con cátedra en el área de Administración de Enfermería, que trata sobre la administración de la asistencia de enfermería, actualmente denominada Gestión de Enfermería, señala que esta estaría orientada, principalmente, a atender las necesidades de seleccionar y adecuar métodos de organización del trabajo, lo que la autora destaca entre paréntesis como SAE^(29)^.

Partiendo de este hilo conductor, un estudio reciente^(25)^ considera que la SAE no se configura como un objeto epistemológico con conexión convergente con la atención directa al paciente, sino que es un concepto que reúne propiedades que le aseguran un campo de conocimiento relacionado con la gestión y/o la administración de los servicios de enfermería, como un modelo o incluso una herramienta para la gestión de la atención^(7)^. Desde esta perspectiva, la SAE abarca dimensiones que trascienden la atención directa al paciente y permite el análisis y la reflexión sobre la eficiencia y la eficacia de las acciones desarrolladas por el equipo de enfermería, con el fin de contribuir a la toma de decisiones en la gestión y las políticas de los servicios, con miras a la excelencia de la atención.

Reconociendo que la SAE es un constructo genuinamente brasileño, aunque a riesgo de que llegue a ser proscrito, es importante destacar que, a la luz de la filosofía de la ciencia— especialmente de la noción de “programas de investigación científica” propuesta por Imre Lakatos^(21)^—, las teorías y los modelos no se abandonan ante las primeras dificultades o críticas. Por el contrario, los grupos que aplican tales estructuras como explicaciones tienden a invertir en su desarrollo y perfeccionamiento, ajustándolas ante las cuestiones planteadas. Abandonar la concepción de una SAE en este momento equivaldría a ceder a la lógica de un descarte prematuro, en detrimento de la maduración de un modelo teórico-empírico con potencial para orientar y fortalecer la gestión de la práctica profesional y científica de la enfermería.

Como ya se ha mencionado, la existencia de constructos potencialmente correlacionados con la SAE en otros contextos internacionales, como el PPM^(17)^, reitera la relevancia de considerar las posibilidades de un modelo singular de Brasil. En cierta medida, un PPM cuenta con atributos convergentes con los fundamentos de lo que sería la SAE, presentándose como una lente ampliada y estructurante, capaz de resignificar la SAE, especialmente en la propuesta de un enfoque sistémico, que integra valores profesionales, procesos asistenciales, relaciones interprofesionales, mecanismos de reconocimiento y modelos de gobernanza.

La estructura de los PPM se compone de componentes esenciales que definen su organización y funcionamiento, tales como: valores profesionales^(26)^, una base teórica y conceptual que sustenta el modelo^(28)^, relaciones profesionales colaborativas, organización de la atención al paciente^(26,28)^, enfoque de gestión participativa^(26)^, liderazgo en todos los niveles de la práctica^(28)^ y mecanismos de reconocimiento y valoración del enfermero^(26,28)^.

Además, los PPM se configuran como dispositivos epistemológicos que otorgan a la enfermería un espacio autónomo, deliberativo y reflexivo, sustentado en valores fundacionales como la dignidad, la responsabilidad y el compromiso ético-estético para una atención de excelencia^(26,28)^. Al instituir una arquitectura conceptual que articula el saber, el hacer y el ser profesional, estos modelos no solo orientan la práctica asistencial, sino que también re-significan los procesos de toma de decisiones en la gestión y las políticas de los servicios de enfermería, contribuyendo a la cualificación de los entornos de atención y a la ampliación de los horizontes de la disciplina^(7)^.

La SAE, tal como se describe en la literatura, se basa en la organización del trabajo del equipo de enfermería^(30)^, articulada estructuralmente en tres pilares fundamentales: método, personal e instrumentos^(25,31)^. Estos pilares tienen como objetivo instaurar una dinámica laboral orientada por un sistema propio, centrado en la gestión de la atención^(10)^, con el propósito de estructurar y mejorar el *saber hacer* del equipo de enfermería en el día a día de la atención.

En este contexto, resulta imperativo que los líderes de enfermería reconozcan que el intercambio y la consolidación de creencias, valores, conocimientos y prácticas no son solo estrategias organizacionales, sino fundamentos ontológicos y epistemológicos para la (re)construcción de la identidad profesional^(7)^. Por analogía con los PPM, la SAE apunta, por lo tanto, a la necesidad de un pensamiento crítico, comprometido con la producción de significados que trasciendan la mera operacionalización técnica y avancen hacia la consolidación de la enfermería como ciencia aplicada y práctica social compleja.

Entendida desde la perspectiva de un PPM, la SAE presenta múltiples atributos: actúa como modelo asistencial, método de trabajo para la organización de procesos, dispositivo organizacional, instrumento de gestión y vía para la materialización de una atención sistematizada, calificada y receptiva a las necesidades en salud^(30)^.

Así, su finalidad epistemológica y operativa reside en ofrecer directrices para la concreción de las acciones de cuidado, orientadas por el PE en los distintos escenarios asistenciales de la gestión del cuidado. Se trata, por lo tanto, de un modelo o herramienta estructurante disponible para el *saber-hacer* profesional, útil para articular teoría y práctica, conocimiento y acción, individual y colectivo, siendo un hito identitario de la enfermería brasileña.

En cuanto a su estructura, coincidimos con los autores^(25,31)^ al reconocer que el pilar “método” puede entenderse a la luz de los valores profesionales de la enfermería, anclados en una epistemología propia—especialmente en las Teorías de Enfermería y en el PE. Este, a su vez, constituye el eje articulador del pensamiento crítico y del juicio clínico del enfermero, orientando al equipo de enfermería en la prestación de cuidados a la persona, la familia, la colectividad y los grupos en situaciones específicas de vulnerabilidad. En el ámbito institucional, este pilar también se relaciona con la forma en que la atención se organiza metodológicamente en el servicio, considerando la misión, la filosofía y los objetivos de la institución de salud, así como la definición de modelos asistenciales que orientan la práctica de la enfermería. Así, el método se materializa en la adopción de referentes teóricos, en la puesta en práctica de las etapas del PE y en la forma en que se planifica, ejecuta y evalúa la atención en el día a día de los servicios.

El pilar “personal” abarca aspectos relacionados con la dotación de personal, la gestión de personas y la formación y el desarrollo de recursos humanos, fundamentales para sustentar una práctica profesional calificada y alineada con las demandas asistenciales^(25,31)^. En esta dimensión se incluyen elementos como la disponibilidad y el dimensionamiento adecuado del equipo de enfermería, las estrategias institucionales de capacitación y educación permanente, la sensibilización del equipo sobre la importancia de la SAE, así como el desarrollo de las competencias clínicas y gerenciales necesarias para la implementación del PE. También se incluyen en este pilar aspectos relacionados con el interés institucional y el compromiso de la dirección en apoyar la implementación de la SAE, garantizando las condiciones organizativas y estructurales para su puesta en práctica.

Por último, el pilar “instrumentos” se estructura a partir de un conjunto de dispositivos normativos y operativos que dan soporte a la práctica de la atención, tales como: manuales de enfermería, reglamentos internos del servicio, protocolos operativos estándar (POE), protocolos asistenciales, *bundles* clínicos, escalas de evaluación y formularios propios de la enfermería. Estos instrumentos no solo ponen en práctica la atención, sino que también contribuyen a su estandarización, seguridad y calidad, en consonancia con los principios de la SAE y respetando los preceptos de la política de seguridad del paciente. También se incluyen en esta dimensión los formularios y sistemas de registro utilizados por el equipo de enfermería para documentar las etapas del PE—como formularios de Recolección de datos, registros de diagnósticos, planificación, prescripciones y evolución de enfermería—, además de instrumentos de evaluación clínica que consideren las características de la clientela atendida y el perfil asistencial de las unidades.

Ante lo expuesto, es posible afirmar que los pilares estructurales de la SAE guardan una estrecha correspondencia con los componentes esenciales de los Modelos de Práctica Profesional descritos en la literatura internacional. El pilar “método” incorpora el PE, elemento central de la práctica clínica, y los marcos teóricos de la disciplina capaces de proporcionar descripciones, explicaciones, predicciones y prescripciones sobre fenómenos y eventos profesionales. El pilar “personal”, al abarcar la gestión de personas, el dimensionamiento y la cualificación del equipo, converge con los componentes relativos al liderazgo, el desarrollo profesional y el entorno de práctica. Por su parte, el pilar “instrumentos”, al reunir protocolos, reglamentos, formularios y otros dispositivos normativos, dialoga con los elementos de los PPM que estructuran la gobernanza, la estandarización de la atención y el apoyo a la toma de decisiones clínicas. Así, se entiende la SAE no solo como un arreglo técnico-operativo, sino como un sistema integrado de fundamentos epistemológicos, organizacionales y prácticos, capaz de potenciar tanto el razonamiento clínico como la gestión de la atención en múltiples dimensiones.

Estas correspondencias evidencian que la SAE, como constructo genuinamente brasileño, puede y debe entenderse como una expresión situada y robusta de un Modelo de Práctica Profesional, reafirmando su relevancia como fundamento organizativo, epistemológico y ético de la atención en Enfermería. De esta manera, se amplía el alcance conceptual y modelar de la SAE más allá de una herramienta organizacional, ofreciendo potencial como estructura representativa de la integración del conocimiento y la práctica.

El enfermero, en su calidad de gestor de la atención, debe pensar en la organización de todo el servicio y orientar la planificación de la atención basándose en el PE y en el razonamiento clínico—entendido como un proceso dinámico que articula la cognición, la metacognición y la toma de decisiones^(27)^. Este pensamiento de naturaleza sistémica, en teoría, promueve la excelencia de la atención capaz de ir más allá del diagnóstico para abarcar las decisiones terapéuticas, el monitoreo y la evaluación continua de esta.

Desde una perspectiva sistémica, la SAE se fortalece como un dispositivo de gestión de la atención en un sentido más amplio y estratégico, vinculado a la toma de decisiones macropolíticas, la planificación, la evaluación de resultados y las políticas institucionales^(8,10)^, estructurando la práctica sobre la base de la autonomía profesional, la competencia técnica y el compromiso con los sujetos de la atención. Esta comprensión exige la capacitación permanente del equipo, el alineamiento con las directrices legales y el reconocimiento de la SAE como expresión de un modelo de práctica fundamentado en valores profesionales y en el ámbito de la ciencia de la enfermería. La articulación entre la SAE, el razonamiento clínico y los fundamentos de los PPM ofrece, por lo tanto, un camino prometedor para la maduración del concepto de SAE en Brasil, contribuyendo a la coherencia teórico-práctica de la atención, a la gestión calificada de los servicios y al fortalecimiento de la identidad de la enfermería como disciplina y campo profesional.

Aunque las reflexiones de este estudio pueden hacer avanzar el conocimiento, existe una limitación fundamental. El estudio tiene limitaciones a la hora de proporcionar aspectos instrumentales específicos para una aplicación distinta de la SAE, lo cual es esencial para garantizar que no se produzcan nuevas superposiciones conceptuales o que se vacíen de contenido los avances obtenidos de la clarificación de la madurez conceptual del PE para la práctica clínica. Sin embargo, esta limitación depende de superaciones continuas en futuras investigaciones que puedan utilizar evidencias aportadas y trasladables de los PPM a la realidad de la implementación de modelos de SAE en Brasil.

## CONCLUSIÓN

A modo de consideraciones finales, la SAE, a pesar de su trayectoria marcada por ambigüedades conceptuales y disputas semánticas, sigue siendo un constructo importante para la organización de la práctica profesional de la enfermería en Brasil. Su reconocimiento como análogo a un Modelo de Práctica Profesional, alineado con los pilares “método”, “personal” e “instrumentos”, permite reinterpretarla no como sinónimo de PE, sino como un modelo de gestión de la atención. La correlación entre estos pilares y los componentes esenciales de los PPM refuerza el potencial de la SAE para estructurar el trabajo del equipo de enfermería de manera crítica, reflexiva e integrada, promoviendo no solo la calidad de la atención, sino también el fortalecimiento de la identidad de la enfermería.

Su permanencia en el campo disciplinario no depende solo de su tradición normativa o de su arraigo cultural, sino de la capacidad de reconfigurarse a la luz de fundamentos epistemológicos sólidos, de la práctica basada en la evidencia y de las demandas contemporáneas de los servicios de salud. Reafirmar la SAE como modelo implica reposicionarla como capaz de sustentar la dimensión macropolítica de la planificación, a fin de orientar un enfoque de gestión participativa y marcado por el liderazgo del enfermero, con el objetivo de contribuir a la excelencia de la atención de enfermería en Brasil en la articulación en red y en las políticas del SUS.

Se concluye reafirmando el compromiso ético de este análisis con la autoría intelectual de las fuentes. La interpretación de las producciones científicas y normativas preservó la integridad de las ideas originales, asegurando que las síntesis elaboradas no distorsionaran el pensamiento de los autores citados.

## Data Availability

Todo el conjunto de datos que apoya los resultados de este estudio fue publicado en el propio artículo.
